# Unraveling False Positives in Unsupervised Defect Detection Models: A Study on Anomaly-Free Training Datasets

**DOI:** 10.3390/s23239360

**Published:** 2023-11-23

**Authors:** Ji Qiu, Hongmei Shi, Yuhen Hu, Zujun Yu

**Affiliations:** 1 State Key Laboratory of Advanced Rail Autonomous Operation, Beijing Jiaotong University, Beijing 100044, China; 2 School of Mechanical and Electronic Control Engineering, Beijing Jiaotong University, Beijing 100044, China; 3 College of Engineering, University of Wisconsin-Madison, Madison, WI 53705, USA; 4 Frontiers Science Center for Smart High-Speed Railway System, Beijing 100044, China

**Keywords:** anomaly detection, visual defect inspection, multilayer perceptron, normalizing flow, object segmentation

## Abstract

Unsupervised defect detection methods have garnered substantial attention in industrial defect detection owing to their capacity to circumvent complex fault sample collection. However, these models grapple with establishing a robust boundary between normal and abnormal conditions in intricate scenarios, leading to a heightened frequency of false-positive predictions. Spurious alerts exacerbate the work of reconfirmation and impede the widespread adoption of unsupervised anomaly detection models in industrial applications. To this end, we delve into the sole available data source in unsupervised defect detection models, the unsupervised training dataset, to introduce a solution called the False Alarm Identification (FAI) method aimed at learning the distribution of potential false alarms using anomaly-free images. It exploits a multi-layer perceptron to capture the semantic information of potential false alarms from a detector trained on anomaly-free training images at the object level. During the testing phase, the FAI model operates as a post-processing module applied after the baseline detection algorithm. The FAI algorithm determines whether each positive patch predicted by the normalizing flow algorithm is a false alarm by its semantic features. When a positive prediction is identified as a false alarm, the corresponding pixel-wise predictions are set to negative. The effectiveness of the FAI method is demonstrated by two state-of-the-art normalizing flow algorithms on extensive industrial applications.

## 1. Introduction

In recent years, visual defect detection has gained significant traction across diverse domains, encompassing quality control in the production of metal products [1,2], printed materials [3], textiles [4], and the inspection of facility and equipment health, such as petrochemical pipelines [5], angle cock alignments [6], and vehicle fault detection [7,8]. When it comes to defect detection in complex systems composed of numerous components, such as railway freight, the potential categories of faults to monitor can number in the thousands. However, real-world applications often involve rare anomalies, leading to a scarcity of positive samples (defects or anomalies) in comparison to negative samples (normal or anomaly-free instances). Consequently, collecting comprehensive samples for infrequent fault categories presents a substantial challenge. Additionally, the costs associated with professional annotation can be prohibitive. As a result, some industrial applications struggle to establish supervised model-training datasets that encompass a wide spectrum of fault types and are confined to monitoring high-frequency faults.

To tackle these challenges, visual anomaly detection (VAD) algorithms have been developed using unsupervised learning approaches [9,10,11,12,13,14,15,16,17,18,19]. These algorithms learn the distributions of anomaly-free instances while modeling anomalies as outliers relative to the distribution of normal ones. Various techniques have been employed in existing VAD methods, including the k-nearest neighbor method [15,20], clustering methods [16,21,22], and normalizing flow (NF) methods [23,24], to model the distribution of normal samples or their extracted features using pre-trained deep networks. The normalizing flow model comprises a sequence of invertible transformations between differentiable manifolds, offering a computationally and memory-efficient solution for similar applications. Normalizing flow-based detectors [17,18,24,25] have achieved state-of-the-art performance on popular pixel-wise AD datasets, such as the MVTec AD [13] and Magnetic Tile Defects (MTD) [26] datasets. However, these models tend to generate false positives, constituting the majority of defect predictions, and fail to meet practical requirements. Similar observations are illustrated in the experiments in References [27,28], underscoring the susceptibility of normalizing flow-based visual defect inspection models to false alarms in complex scenarios.

Despite these efforts, the fundamental assumption that feature vectors of normal samples follow a multivariate normal distribution may not always be valid. This inconsistency can be attributed to the limited scale of training samples or the extracted features, which may not capture all possible variations within normal scenarios. In other words, multivariate normal distribution may not accurately model the distribution of anomaly-free instances in intricate industrial systems. Recent methods have attempted to introduce a small number of defect samples into the anomaly-free training dataset using open-set settings to improve detection performance in complex industrial scenarios [29,30]. However, this approach compromises the unsupervised learning nature of the model.

To this end, we propose an efficient two-stage approach to enhance the defect performance of unsupervised anomaly detectors. We supply the baseline detection model with the post-processing False Alarm Identification (FAI) module to identify false positives. The key technical contributions of this work include the following:

(1) **Semantic Feature Capture**: We introduce a two-stage approach that is adept at capturing semantic features related to potential false alarms at the object level. The FAI model leverages the anomaly score distribution derived from the baseline detector, which is trained on anomaly-free training images. A detailed exploration of this method can be found in Section 3.2.
(2) **Multilayer-Perceptron-Based FAI**: We develop an innovative FAI algorithm based on a multilayer perceptron. This algorithm is specifically tailored to identify false positives from positive prediction patches generated by the baseline detector and subsequently updates both pixel-level and image-level predictions. The intricacies of this algorithm are elaborated upon in Section 3.3.
(3) **Adaptive Augmentation Strategy:** We devise an adaptive augmentation method to generate baseline-detector-dependent training samples for the FAI model. This method operates on the anomaly-free training dataset, effectively creating a balanced set of training samples for the FAI model. We further provide a simulation experiment to demonstrate the discrimination ability of the multilayer-perceptron-based FAI using semantic features and the augmentation strategy. The details of this strategy are discussed in Section 3.4.

Furthermore, the effectiveness of our proposed approach is extensively validated through comprehensive experiments conducted on industrial applications. These experiments provide insights into the elimination of false positives generated by two advanced unsupervised anomaly detection models at both image and pixel levels, as comprehensively presented in Section 4.

The remainder of this paper is structured as follows: Section 2 presents preliminary information and related work on unsupervised visual defect detection models. Section 2.1 summarizes the relevant research, while Section 2.2 outlines how the normalizing flow model works in visual defect detection tasks, from deep features extracted from a pre-trained deep model to the anomaly map. Building upon the normalizing flow models, we introduce the details of the proposed methodology in Section 3. In Section 4, we present experimental results, demonstrating that our proposed model enhances advanced NF defect detectors in two industrial applications. Finally, the conclusion is summarized in Section 5.

## 2. Preliminaries and Related Work

In this section, we provide an overview of relevant prior work. Section 2.1 introduces the concept of unsupervised anomaly detection, a process that entails constructing a model of normality based on an anomaly-free training dataset and employing it to identify deviations in new instances. Moreover, it elucidates the functioning of anomaly detection at both image and pixel levels. In Section 2.2, we delve into the fundamental principles of normalizing flow models, discussing their application in the context of anomaly detection tasks. We also briefly address the concern of false alarms and allude to the necessity of post-processing techniques for mitigating this issue.

### 2.1. Unsupervised Defect Detection

Unsupervised Anomaly Detectors (UAD) acquire a model of normality from the anomaly-free training dataset and make predictions regarding the class of a new instance by assessing deviations from the learned distribution. Consequently, unsupervised anomaly detection offers a solution for defect inspection without an exhaustive data setup. Prominent unsupervised anomaly detection models, such as Variational Autoencoders (VAEs) [31,32,33,34,35], Generative Adversarial Networks (GANs) [36,37,38,39,40,41,42,43], and normalizing flows [12,14,17,18,23,44,45,46,47,48], find extensive application in visual defect detection tasks.

Visual anomaly detection presents challenges, especially when dealing with high-dimensional data and complex applications at the pixel level, i.e., object segmentation. In the case of an image sample, the defect detector generates prediction scores at both image and pixel levels. Pixel-level prediction scores form the anomaly map of the instance. The image-level prediction score and the anomaly map turn into the image-level classification result and the segmentation mask through binary thresholding, respectively.

In industrial visual defect inspection tasks, promising unsupervised methods encompass VAEs [33,34], GANs [36,37], and normalizing flows [14,17,18,19,46]. These methods often benefit from deep features obtained from pre-trained backbones such as ResNet [49], Wide ResNet [50], and ShuffleNet [51] on large-scale image datasets like ImageNet. Performance differences predominantly arise during the density distribution estimation phase, in which a transformation is constructed to map image intensities or deep features into prediction scores. Please refer to References [9,10,11] for comprehensive comparisons and surveys.

One distinguishing characteristic of normalizing flows is their tractable density modeling [19,23]. Unlike implicit density generative models or approximate density estimation, normalizing flow models explicitly construct expressive density distributions over continuous random variables through bijective mappings. In contrast to the study by Qiu et al. [45], our focus lies on this unique feature of NF models, and we devise a False Alarm Identification algorithm tailored for their anomaly maps. The FAI model delivers competitive performance in complex scenarios. A simulation experiment and parameter study with various augmentation settings further illustrate the classifier’s discrimination ability and the sample synthesis strategy within the FAI algorithm.

### 2.2. Normalizing Flow-Based Anomaly Detection

The normalizing flow model is constructed as a sequence of invertible transformations between differentiable manifolds, which are diffeomorphisms [9,18]. Consider a vector $z_{0}$ with probability distribution $p_{z_{0}}\left(z_{0}\right)$ and let $f:Z_{0}\to Z_{1}$ be one-to-one, continuously differentiable mapping from the differentiable manifold $Z_{0}$ to another differentiable manifold $Z_{1}$, also with continuously differentiable inverse mapping. We can express this relationship through the integral formula

(1) $$\int_{z_{0}}p_{z_{0}}\left(z_{0}\right)dz_{0}=1=\int_{z_{1}}p_{z_{1}}\left(z_{1}\right)z_{1}$$

Consequently, the probability density distribution of the resulting variable is given by

(2) $$p_{z_{1}}\left(z_{1}\right)=p_{z_{0}}\left(z_{0}\right)\left|\frac{dz_{0}}{dz_{1}}\right|=p_{z_{0}}\left(z_{0}\right)\left|\frac{\partial f^{-1}\left(z_{1}\right)}{\partial z_{1}}\right|$$

Let $J_{f^{-1}}\left(z_{1}\right)$ represent the Jacobian matrix of $f^{-1}$ at $z_{1}$:

(3) $$J_{f^{-1}}\left(z_{1}\right)=\frac{\partial f^{-1}\left(z_{1}\right)}{\partial z_{1}}$$

And the Jacobian matrices of f and $f^{-1}$ are related by

(4) $$detJ_{f^{-1}}\left(z_{1}\right)=detJ_{f}{\left(z_{0}\right)}^{-1}$$

Therefore, we may have Equation (2) as

(5) $$p_{z_{1}}\left(z_{1}\right)=p_{z_{0}}\left(z_{0}\right)\left|detJ_{f^{-1}}\left(z_{1}\right)\right|=p_{z_{0}}\left(z_{0}\right)\left|detJ_{f}\left(z_{0}\right)\right|$$

If we denote $f_{k}:Z_{k-1}\to Z_{k}$ with $f:Z_{0}\to Z_{k}$, the initial density distribution $p_{z_{0}}\left(z_{0}\right)$ flows through these mappings, resulting in expansions or contractions. The final density distribution $p_{z_{K}}\left(z_{K}\right)$ is generated through the repeated application of the rule for the change in variables

(6) $$f=f_{K}\circ \cdots \circ f_{1}$$

Subsequently, the distribution is built through successive applications of the chain rule for K transformations:

(7) $$log\left(p_{z_{K}}\left(z_{K}\right)\right)=log\left(p_{z_{0}}\left(z_{0}\right)\right)-\sum_{k=1}^{K}log\left(det\;J_{f_{k}}\left(z_{k-1}\right)\right)$$

In anomaly detection tasks, the initial variable $z_{0}$ typically represents the deep features extracted from a pre-trained deep model, while the target $z_{k}$ corresponds to the anomaly score obtained as the final output of the normalizing flow model. To model the target distribution $p_{z_{K}}\left(z_{K}\right)$ into a multivariate normal distribution, a normalizing flow model is trained by minimizing divergence or discrepancy, as outlined in Reference [23].

It is standard practice for anomaly detection algorithms to produce two pivotal outputs for visual defect inspection tasks: an image-level prediction score and an anomaly map comprising pixel-level prediction scores. The image-level prediction score functions as an indicator of the classification outcomes for a given test image, efficiently distinguishing between a defect or defect-free state through binary image classification. Conversely, the anomaly map represents the target distribution $p_{z_{K}}\left(z_{K}\right)$, which is subsequently converted into the predicted segmentation mask by a classification threshold τ.

Although trained GAN models yield promising performances in relative vision tasks, instability in their training process increases from complex hyperparameter tuning. Different from the discriminative networks of GANs and the approximate inferences of VAEs, bijective mappings give rise to compact and effective architectures of normalizing flow models, which ensures their advanced detection performances at the pixel level.

In the absence of defect-specific information, unsupervised anomaly detectors often classify outliers as defects. Consequently, when dealing with intricate defect definitions and noisy backgrounds, the performance of these detectors tends to deteriorate. Among various anomaly detection models, normalizing-flow-based models stand out due to their efficiency in tractable likelihood estimation. However, as demonstrated by experiments [27,28], normalizing flow models tend to generate false alarms, particularly in complex scenes. This observation has prompted us to explore post-processing techniques for mitigating false positives.

## 3. Proposed Method

In this section, we present a systematic approach to enhancing unsupervised defect inspection by addressing false alarms and efficiently identifying them through a combination of structural and data synthesis strategies. We begin by discussing the difficulties faced by unsupervised defect inspection in complex industrial settings, as outlined in Section 3.1.

To overcome these challenges, we present a two-stage workflow for reclassifying false alarm patches generated by the baseline UAD model, as detailed in Section 3.2. The core module of our false alarm elimination, a multilayer perceptron structure, is designed to identify detector-dependent false alarms by analyzing semantic features. Section 3.3 provides comprehensive insights into this structure and explains how to process identified false alarms.

One of the primary challenges in training the False Alarm Identification module is the absence of true positive samples. To tackle this issue, we introduce a sample synthesis strategy, elaborated in Section 3.4, which generates true positives and emulates the effects of adverse factors that contribute to false alarms. Additionally, we illustrate the discriminative capabilities of the proposed method through a simulation experiment.

### 3.1. Insights from Visual Defect Detection Training Data Analysis

In visual defect inspection, the performance degradation of unsupervised defect inspection algorithms in complex industrial settings is attributed to two fundamental challenges: the inherent complexity within “normal” data and the complexity between “normal” and “defect”.

Firstly, a significant challenge arises when dealing with a diverse array of images capturing complex systems with multiple components. The crux of this challenge becomes evident when we delve into the density probability distributions of prediction scores within the anomaly-free training dataset.

Ideally, prediction scores for anomaly-free images should consistently remain below a predefined threshold, typically set to 0.5. This threshold is employed to distinguish between anomaly-free pixels and those exhibiting defects. However, in anomaly-free samples from real-world scenarios, certain patches, adhering to specific semantic rules, tend to generate high responses within the anomaly map. In some cases, these high responses surpass the predetermined threshold (τ), resulting in false positives even within the training dataset.

It is crucial to note that the training dataset consists of anomaly-free images, yet it encompasses a multitude of distinctive features.

**Figure 1:**
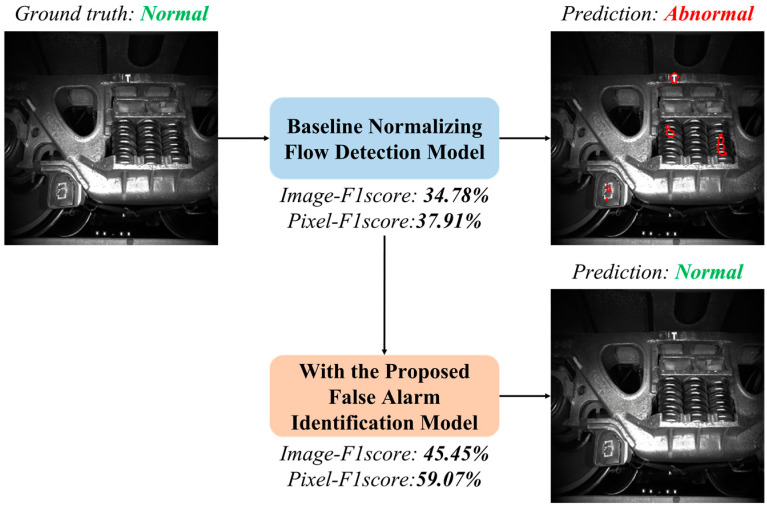
Defect inspection results of a state-of-the-art normalizing-flow-based defect detection model [18] without and with the proposed model on side frame images of freight trains. An example image misclassified by the baseline model is presented. With the proposed false-positive filter, false-alarm pixels on the example are eliminated and the image-level prediction class is correct. The proposed algorithm improves the performance at both metrics of the pixel level.

Figure 1 depicts an image captured by the Trouble of Moving Freight Car Detection System (TFDS), an uninterrupted visual sensing method for monitoring freight trains in operating mode. In freight fault detection tasks, variations in appearance (image intensity) can be attributed to a myriad of external factors, such as fluctuations in lighting conditions, cluttered backgrounds, and intrinsic factors like product batches, gradual aging during service, the presence of hand-written stamps, and material characteristics. These factors contribute to a diversity of image intensities or feature heatmaps within the corresponding subregions. Furthermore, this diversity extends to various locations within the image, each corresponding to different components of the monitored complex system.

Therefore, FastFlow, a state-of-the-art normalizing-flow-based defect detection model, erroneously categorizes this image as an anomaly due to unusual large-area reflections, leading to a false alarm. Specifically, we can discern that the lower-left corner of the image captures the wheels, where lighting reflections and handwritten stamps may be present. Conversely, the lower middle section of the image corresponds to the background, exhibiting varying intensities over a wide range. This intricate interplay of complex factors inevitably leads to misclassification, particularly when the training dataset consists of a limited number of samples. In such scenarios, false alarms stem from the model’s inclination to overfit dominant patterns within anomaly-free ranges. Addressing this issue is challenging, especially for deep CNN models operating with a limited number of instances.

The second challenge arises from the inherent delineation between “normal” and “defect”. Unsupervised defect inspection algorithms often classify outliers as defects, but the definition of defects is intrinsically dependent on domain-specific knowledge in specific applications.

On one hand, when considering the temporal dimension and excluding external factors in the image acquisition process, the appearance of a specific region in the image often exhibits gradual changes. Yet determining anomalies in these regions can be arbitrary without practical criteria. For instance, in freight train fault detection, the permissible threshold for wheel tread wear is 8 mm. However, assessing this wear limit is challenging through visual image analysis due to factors like shooting angles and occlusions. The representation of its thickness in the image typically encompasses only a few pixels. Even professional vehicle inspectors may dispute some cases approaching the critical limit. Therefore, UAD models lacking domain-specific knowledge face increased difficulty in handling deformation-related faults and exceedance-related anomalies involving gradual changes, resulting in a deteriorating performance in classifying relevant pixels.

On the other hand, some types of defects may not be perceptible solely from visual images in practical scenarios. For instance, determining whether rust on the brake beam bracing strut of a freight train constitutes a crack defect or not may necessitate further actions, such as erasing traces and conducting physical inspections. This disparity between classification categories and visual observations challenges the distinction between “normal” and “defect”, hindering the establishment of a comprehensive anomaly-free training dataset.

In conclusion, the discriminative capabilities of unsupervised detectors often encounter challenges when faced with intricate classification boundaries distinguishing defects from defect-free instances in noisy and complex industrial applications. Without comprehensive additional annotations, this problem can hardly be resolved solely through deep networks with extensive parameters. Our objective is to develop an algorithm capable of identifying samples classified as anomalies by the baseline UAD model and determining whether they are false alarms that should be reclassified as negative instances.

### 3.2. Anomaly-Free Training Dataset and the False Alarm Identification Method

In the absence of introducing additional samples, the only available data source is the anomaly-free training dataset. As a post-processing approach, we delve into the performance of the trained normalizing-flow-based detection model on the anomaly-free training dataset. Since the inference of the normalizing flow method relies on a sequence of invertible transformations, we further deduce that factors causing high responses in the unsupervised training dataset will similarly lead to high responses in the test dataset, as evidenced by similar prediction score distributions on the anomaly maps. High responses in the unsupervised training dataset suggest that these factors are more likely to induce false positives in the test dataset. Therefore, the post-processing method proposed in this paper is based on an assumption: high responses in the anomaly maps of the test dataset, resembling the pattern of high prediction score regions in the training dataset’s anomaly maps, indicate false alarms.

**Figure 2:**
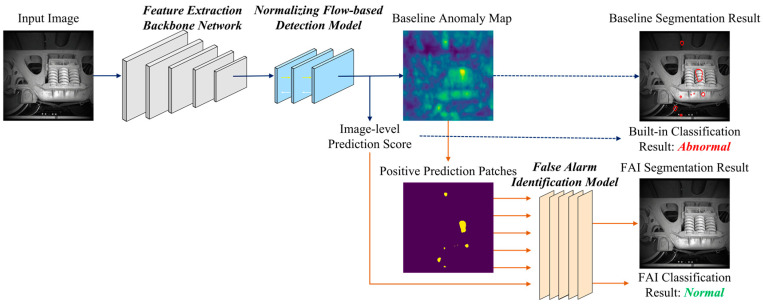
The two-stage false-positive filter workflow for visual defect inspection applications. Blue arrows describe the baseline normalizing-flow-based anomaly detection process currently regarded as the first built-in stage. Orange arrows present the second stage, where our proposed FAI model makes predictions based on the outputs of the first stage.

Based on this assumption, we introduce the False Alarm Identification (FAI) method, as shown in Figure 2.

Normalizing-flow-based anomaly detectors address defect inspection by framing it as an out-of-distribution problem. More precisely, they seek to model the anomaly scores associated with “normal” pixels as a multivariate Gaussian distribution. However, this assumption encounters challenges in complex defect detection scenarios in which the “normal” patterns deviate from the centroid of the normal distribution, leading to the generation of false alarms. Consequently, our proposed workflow involves a two-phase process initially employing a normalizing flow model for outlier detection, followed by the re-classification of positively predicted patches to identify and differentiate false alarms based on semantic features.

The FAI method operates at the “object level”, achieved through binary masking and connected component segmentation techniques. This term, “object level”, is a fundamental concept within a hierarchical framework encompassing three granularity levels: pixel, object, and image; these levels correspond to specific vision tasks—image segmentation, object detection, and image classification, respectively. This concept is also commonly referred to as the “region level” in the related literature. Our choice of “object level” effectively captures the inherent semantic attributes found in patches within defect detection images, often aligning with specific components of the inspected object or system, making it the most suitable term for our context.

As depicted in Figure 2, the proposed method focuses on addressing the positive prediction patches generated by the normalizing flow method to mitigate false alarms. The training and testing procedures of the baseline normalizing flow model, constituting the initial detection phase, remain unchanged. During the training phase, the proposed FAI model learns the distribution of high-score patches from the anomaly maps of the anomaly-free training dataset, leveraging the pretrained NF model. In the testing phase, the FAI model identifies these learned patterns from the positive prediction patches of the NF model based on their semantic characteristics (Section 3.3).

Acknowledging that the quantity of false positives might not be adequate for our training requirements, we introduce corresponding data augmentation methods in Section 3.4. These methods are designed to facilitate the training of the FAI model without additional labeled samples.

### 3.3. The Multilayer-Perceptron-Based False Alarm Identification

To design a lightweight and effective False Alarm Identification system, we aim to enhance the reclassification efficiency from two key perspectives: network architecture and semantic features.

From a network architecture standpoint, we employ a multilayer perceptron (MLP) to capture detector-dependent false alarms, using anomaly maps derived from training instances. While MLP networks may lack the depth of Deep Neural Network (DNN) models, their multiple hidden layers offer commendable performance with significantly lower computational demands and reduced training sample requirements. MLP networks excel in capturing and modeling non-linear relationships in complex classification tasks, outperforming other lightweight classifier models such as Support Vector Machines (SVMs).

In the processing of object-level data, our primary focus is semantic features. Semantic features encapsulate high-level information that transcends pixel-level characteristics. This prioritization proves to be particularly advantageous, especially in complex defect detection images where physical constraints, including object location, size, shape, color, and intensities, play a pivotal role in distinguishing specific components of the monitored target.

Through semantic features with attributes encompassing dimensions, location, color histograms, and more, we provide meaningful characteristic summaries of positive patches. This approach leverages this valuable information, resulting in a heightened understanding of false positives and their relevance within the specific application.

Note that flow-based models and pre-trained networks have inherent limitations when dealing with unsupervised learning; as demonstrated in the extensive literature analysis, they struggle to effectively model normal states in complex scenes. In reality, the ability of MLP models to detect these challenging samples is not solely attributed to their architecture but rather to the introduction of a more diverse set of discriminative angles. Essentially, we extend the re-classification by leveraging object-level semantic features in conjunction with the simplicity and discriminative power of MLP models. This additional angle aids in the efficient re-classification of defects by the baseline detector in specific application contexts.

From the normalizing flow model, positive prediction patches can be obtained. Let us define $x_{m}$ as the input capturing the object-level physical constraints of a defect proposal denoted as χ. This input corresponds to a subregion on the image that aligns with a positive prediction patch on its anomaly map. We further define y as the output of the FAI model, which indicates the filtering result of this positive prediction patch.

The designed multilayer perceptron comprises two hidden layers, each having L and N neurons, respectively. The output of the first layer neurons can be represented as

(8) $$z_{l}=f_{1}\left(\sum_{m=1}^{M}\omega_{lm}x_{m}+\theta_{l}\right)\;\;\left(l=1,\ldots ,L\right)$$

where the activation function $f_{1}\left(\cdot \right)$ corresponds to the linear rectification function. The parameters $\omega_{lm}\left(l=1,\ldots ,L;m=1,\ldots ,M\right)$ and $\theta_{l}$ are the weights and bias of the first-layer neurons, respectively. Assuming the second matrix of parameters $\omega_{nl}\left(n=1,\ldots ,N;l=1,\ldots ,L\right)$ and the offsets in the last layer $\theta_{n}$, we can describe the output vectors of the second hidden layer as

(9) $$z_{n}=f_{1}\left(\sum_{l=1}^{L}\omega_{nl}z_{l}+\theta_{n}\right)\;\;\left(n=1,\ldots ,N\right)$$

Consequently, we can express an analytical function that maps from $x_{m}$ to y as follows:

(10) $$y\left(x_{m}\right)=f_{2}\left(\sum_{n=1}^{N}\omega_{1n}f\left(\sum_{l=1}^{L}\omega_{nl}f\left(\sum_{m=1}^{M}\omega_{lm}x_{m}+\theta_{l}\right)+\theta_{n}\right)+\theta \right)$$

where $f_{2}\left(\cdot \right)$ is a sigmoid function.

To evaluate the degree of correspondence between the ground truth and the output false-alarm score, we employ the L2 distance. The loss function for our model is based on the mean squared error. We employ the Adam optimization algorithm, which is well-suited for scenarios with potentially noisy or sparse gradients.

The output generated by the MLP-based False Alarm Identification model, denoted as $y\left(x_{m}\right)$, corresponds to the false-alarm likelihood of the candidate positive patch χ. This likelihood serves as an indicator of the patch’s classification result. To modify the baseline prediction of the normalizing flow model, we encounter three essential parameters: the false-alarm likelihood threshold ($\tau_{FAI}$), the pixel-level anomaly score factor ($r_{p}$), and the image-level anomaly score factor ($r_{i}$).

If a defect proposal exhibits a high false-alarm likelihood that surpasses the threshold ($\tau_{FAI}$), it is categorized as a false alarm. Subsequently, adjustments are made to the predictions generated by the normalizing flow model at both the pixel level and the image level. At the pixel level, the anomaly scores of the pixels corresponding to the candidate positive patch χ are multiplied by a factor $r_{p}$ that is less than 1. This step is taken to eliminate the false alarms within the segmentation map. Furthermore, the image-level prediction score is reduced proportionally by the ratio of pixels covered by the patch χ to the total number of pixels in the image. This reduction factor is determined by the image-level anomaly score factor $r_{i}$.

### 3.4. An Adaptive Sample Synthesis Strategy for Industrial Applications

As a binary classifier, the primary challenge faced by the proposed FAI module arises from the scarcity of training data for a single class. In its role as a re-classification model for positive candidates, the training phase of the FAI model requires the availability of both false positives and true positives. False positives are obtained from patches in the training images with high prediction scores. However, true positives, corresponding to defect annotations, are notably absent.

To address this challenge, we introduce the generation of true positives using a random approach for training the FAI model. It corresponds to a physical scenario in which defects can appear randomly within the image capture range, occupying any position, or size, and exhibiting various image intensities. The fitted true positives may even share similar semantic features with false positives. This assumption is logical and aligns with the analysis presented in Section 3.1, as true positives could indeed exhibit similar characteristics to false alarms, making them challenging to distinguish through simple visual inspection.

**Figure 3:**
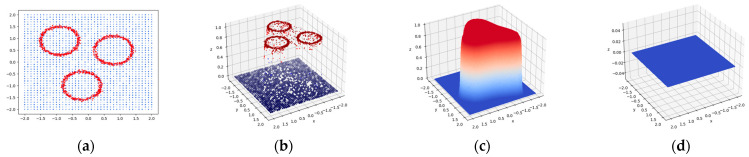
Visualization training samples and model outputs (red, false alarm; blue, defect). Circle points in (**a**) visualize the radii and locations of training samples. The colored 3D points in (**b**) depict their classification results from the FAI model. (**c**,**d**) The output surface at radius r=1 and r=20.

To further illustrate the discriminative capabilities of the proposed method under the sample generation strategy, we conduct a simulation experiment. Figure 3 illustrates the discriminative capabilities of the FAI model in a simulated experiment. In this experiment, 1681 false alarm training instances are distributed around three hollow circles, each having varying radii uniformly distributed in [0.1, 4] (corresponding to the bounding box dimensions of the patches). Defect instances are generated in the same quantity as false positives. Each defect sample is uniformly placed within the image, with the radii following a different uniform distribution in the range [0.5, 20] compared to the false positives. This complex nonlinear structure presents significant challenges for differentiation. By selecting the radii and location as discriminative attributes, these instances are transformed into 3D semantic features, comprising a 1D radius and a 2D center location, which are then employed to train the FAI model.

Binary testing samples are generated from the same strategy. Figure 3b depicts their classification results in which the proposed model successfully filters out complex false alarms with few mistakes. Hard samples like defects located in the central regions of hollow circles are classified correctly. In 50 tests, the accuracies of all models (100 training epochs) reach a minimum of 95% in such a complex and noisy setting. Figure 3c,d further demonstrate the influence of the area dimension. Predictions for samples with uniform areas at r=1 are prone to high likelihoods. Output scores for large-area samples at r=20 are near zero. This proves the discrimination ability of the proposed FAI model and the effectiveness of training sample synthesis approach.

This experiment offers empirical evidence of the FAI model’s proficiency in addressing challenging scenarios in which true positives exhibit characteristics resembling false alarms. To some extent, defect instances are introduced to mitigate the risk of overfitting in the FAI model. In the context of post processing for defect detection tasks, these instances represent artificial semantic feature vectors generated by a random defect sample generator. The dimension of these vectors is determined by specific physical constraints.

The issue with synthesized false positive instances lies in the potential insufficiency of their quantity for effectively training the FAI model. We must investigate the factors contributing to the occurrence of false positives. False positives can be attributed to various factors encompassing lighting conditions, the optical characteristics of components, and the backdrop against which the inspections are conducted. Furthermore, the life cycle of the components can significantly impact the defect inspection process in maintenance management. Brand-new components may exhibit variations compared to those that have been in operation for an extended period, which might show signs of slight aging or accumulate dust while remaining functional. Therefore, it is important to note that a consistent causal factor can result in similar effects, leading to a common pattern among false positives.

Assuming that adverse factors responsible for false positives in the testing dataset are also present in the training dataset, we employ adaptive binary thresholds to identify potential false positives even within correctly predicted images. As mentioned earlier, the normalizing flow method generates an anomaly map for each image which comprises prediction scores that reflect the proximity of each pixel to the learned normal distribution derived from the anomaly-free dataset. Although predictions for a training image may be accurate using a standard threshold, the model’s robustness depends on the values of these prediction scores. Given that the influence of adverse factors persists, the distribution of anomaly values becomes an indicator of the defect detection model’s discriminative capability. Lowering the binary threshold would result in regions with relatively high scores being identified as defect proposals, mimicking the process that leads to false alarms during testing, where the model’s ability to discriminate is compromised for unknown images.

**Figure 4:**
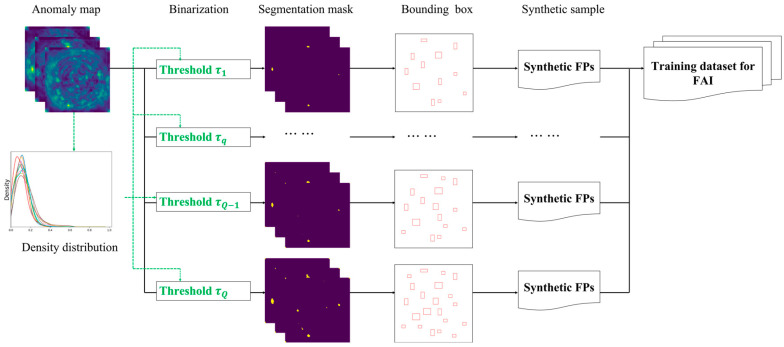
False-positive sample synthesis workflow on the anomaly-free dataset.

Hence, we introduce minor adjustments to the binarization threshold of the anomaly, thereby generating original defect samples, as depicted in Figure 4. The extent of these adjustments depends on the conventional binary threshold and the distribution of anomaly scores.

To further increase the sample quantity, fundamental augmentation strategies encompass cropping, zooming, and noise addition. In the augmentation process, the synthesis of defects aligns with the general practices employed in conventional tasks. However, when generating false positives, it is essential to adhere to the specific rules dictated by the application, which may involve factors like the shooting method and the characteristics of the target. For example, when augmenting samples for inspecting bottle manufacturing, particular emphasis is placed on preserving the central symmetry attribute.

## 4. Experiments

In Section 4, we conduct experiments to evaluate the performance of the proposed false-alarm elimination method using two advanced normalizing flow algorithms. We maintain consistent parameters for the baseline algorithms throughout the comparative experiments, allowing us to assess the differences in performance between the baseline NF detection models and their revised versions with the integrated FAI model.

Section 4.1 and Section 4.2 provide an introduction to the baseline NF detection models, the experimental datasets, and the evaluation metrics employed in our study.

In Section 4.3, Section 4.4 and Section 4.5, we delve into the utilization of nine binary thresholds for sample synthesis (ranging from 0.4 to 0.52) and the use of semantic features derived from the joint distribution of the 2D bounding box size and 2D central location in different application scenarios. Section 4.3 includes an ablation experiment that comprehensively compares the impact of semantic feature selection and binary threshold settings. In these experiments, the false-alarm likelihood threshold $\tau_{FAI}$ in the FAI module is consistently set to 0.5. The pixel-level anomaly score factor $r_{p}$ is set to 0.5. The image-level anomaly score factor $r_{i}$ is adjusted to 2 for Cflow experiments and 3 for Fastflow experiments.

### 4.1. Baseline NF Detection Models

The proposed model is evaluated on two advanced NF defect models, namely Cflow [17] and Fastflow [18].

Cflow introduces a conditional normalizing flow framework for anomaly detection with localization. It comprises a discriminatively pre-trained encoder followed by multi-scale normalizing flow decoders. The encoder extracts multi-scale pyramid features to capture global and local semantic information. Pooled features are further processed by decoders separately to estimate the anomaly maps of encoded features.

Fastflow designs a two-dimensional normalizing-flow-based probability distribution estimator that acts as a plug-in module with deep feature extractor backbones for unsupervised anomaly detection and localization. In the training phase, Fastflow learns to transform the input visual feature into a tractable distribution. In the inference phase, it assesses the abnormal likelihood pixel-wise.

In the following experiments, input images are resized into the specified image resolution 256×256. Their estimated multi-scale anomaly maps are upsampled to the input size and combined to produce the anomaly map. To enhance the diversity, Fastflow is configured with ResNet18, while Cflow utilizes Wide ResNet50. Table 1 presents the relevant configuration details for CFlow and Fastflow.

### 4.2. Experimental Datasets and Evaluation Metrics

Considering the wide-ranging applications of defect inspection within the manufacturing and maintenance industry, we employ industrial defect detection scenarios drawn from two primary sources to validate the effectiveness of our proposed methodology.

We leverage the MVTec AD anomaly detection dataset, a well-established and widely used resource in industrial defect inspection. Our experiments encompass all 15 categories available in this dataset. To offer a more detailed view of our approach in action, we focus on the bottle quality inspection scene, which serves as a representative example of a scenario characterized by stable lighting conditions and simple structural targets. This task comprises 209 training images and 83 test images, all maintaining a consistent resolution of 900 × 900 pixels.

The TFDS-SF inspection dataset, on the other hand, represents a more complex and challenging defect inspection scenario. It consists of TFDS side frame images taken outdoors with background disturbances. Unlike the previous scene in which apparent differences indicated defects, the appearance changes of the image patches in this application come from internal or external factors. This dataset comprises side frame images of different vehicles in a freight train, resized to a resolution of 1024 × 1024. The TFDS-SF inspection dataset includes 180 anomaly-free training images and 47 testing images of three fault types.

We analyze the experimental results from three perspectives: whether the training set of this scenario conforms to the assumption, performance metrics, and the visualization of false alarm identification of the FAI model.

To ensure comprehensive assessments, we utilize the pixel-wise outputs of these algorithms and compare them with the baseline algorithms using four metrics: the image AUROC (area under the receiver operating characteristic), the image F1-score, the pixel AUROC, and the pixel F1-score.

All these performance metrics are evaluated based on a confusion matrix which consists of four classes: true positive (TP), false positive (FP), false negative, and false negative (FN). The AUROC is calculated as the area under the ROC curve, which demonstrates the trade-off between the true positive rate and false positive rate across various decision thresholds. For unsupervised binary image-wise or pixel-wise classification tasks, the AUROC provides valuable insights into a model’s discrimination ability, considering potential data imbalances, especially at the pixel level.

In addition, the F-Measure leverages precision and recall into a single measure that captures both properties. Their formulae are as follows:

(11) $$precision=\frac{TP}{TP+FP}\times 100\%$$

(12) $$recall=\frac{TP}{TP+FN}\times 100\%$$

(13) $$F1\text{-}score=2\times \frac{precision\times recall}{precision+recall}\times 100\%$$

### 4.3. Experimental Analysis on Bottle Quality Inspection

#### 4.3.1. Anomaly Maps on the Training Datasets

**Figure 5:**
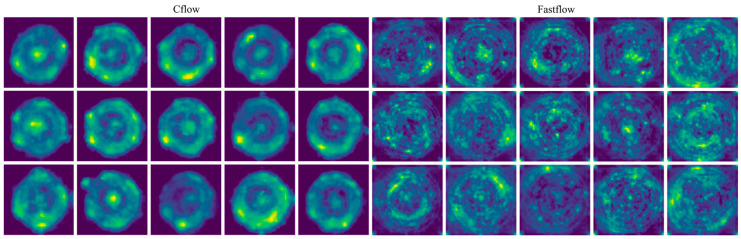
Anomaly maps of Cflow (**left**) and Fastflow (**right**) on the anomaly-free training instances of the bottle quality inspection scene.

Figure 5 illustrates the anomaly maps generated on the training dataset by the two trained detectors, Cflow and Fastflow, employing identical color normalization settings. A significant observation is the distribution and characteristics of highlighted regions. Fastflow’s anomaly maps exhibit more dispersed highlights, with smaller patch areas but a higher quantity compared to Cflow.

#### 4.3.2. Performance Comparisons

Table 2 provides a comprehensive comparison of all detection metrics for the two NF models before and after the incorporation of the FAI module. Given the simplicity of the lighting conditions in the Bottle Quality Inspection dataset, all models achieve nearly perfect performance in terms of the image AUROC scores and F1-scores.

**Figure 6:**
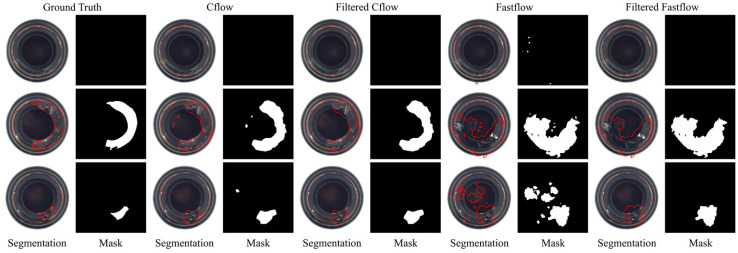
Pixel-level fault detection results of Cflow (**medium**) and Fastflow (**right**) on the bottle quality inspection scene: segmentation boundaries and masks.

Performance metrics at the pixel level pose a greater challenge and offer a finer-grained assessment of a detectors’ detection capabilities. To gain a deeper appreciation of the importance of these performance metrics and the enhancements achieved by our post-processing technique, Figure 6 showcases representative visualization results. The first row displays an example of a negative test image, while the subsequent two rows showcase examples of positive test images.

#### 4.3.3. Visualization of Segmentation Results

The segmentation masks generated by the Cflow and Fastflow models exhibit semantic characteristics similar to the anomaly maps of training images, as shown in Figure 5, particularly in terms of size and scale. The false positive predictions produced by Cflow are concentrated near the circular bottoms of the bottles and are relatively fewer in number. In contrast, the false positive predictions generated by Fastflow are more fragmented in terms of size and exhibit greater variability in their spatial distribution. This performance confirms the validity of the assumption made in this study regarding the consistency between the training and testing datasets in this specific scenario.

In particular, the first row in Figure 6 illustrates an anomaly-free image incorrectly classified as positive by Fastflow. This misclassification explains why Fastflow achieves a perfect image-level AUROC but exhibits an F1 score lower than expected for a defect-free image (with image-level prediction scores slightly exceeding 0.5). After applying the FAI method, the false-positive patches are successfully identified, resulting in a decrease in the image-level prediction score. This correction leads to the Fastflow model achieving a perfect image F1-score when combined with the FAI model.

The second and third rows in Figure 6 demonstrate the impact of the filtering process on test images with defects. The FAI algorithm effectively eliminates numerous small false alarm patches, resulting in improvements in pixel-level metrics for both models. With the FAI model, the image-level F1 score of Fastflow increases by 2.4%, and the pixel-level F1 score increases by 1.7%. While these changes may not appear significant in the numerical values of performance metrics, Figure 6 reveals the substantial elimination number of false alarms of the FAI model at the object level.

The experiments conducted on bottle inspection datasets are designed for controlled environments with well-defined surveillance targets. The FAI algorithm effectively eliminates small false positives and provides slight performance enhancements for both trackers.

### 4.4. Comparative Experiments on MvTec AD Dataset

In this subsection, we provide a comprehensive summary of the impact of our FAI model on two NF detectors across the remaining 14 categories within the MVTec AD dataset. Given the substantial number of categories involved, we refrain from a detailed individual analysis of anomaly maps for the training dataset, instead focusing on listed performance metrics. Additionally, we present segmentation visualizations to offer representative insights into these experiments.

#### 4.4.1. Performance Comparisons

The experimental comparisons across 14 categories are summarized in Table 3 and Table 4, distinguishing between pixel-level and image-level performance metrics.

As shown in Table 3, the incorporation of the FAI module yields noticeable enhancements in both pixel-level detection metrics, the AUROC and the F1-score, for the Cflow and Fastflow models across almost all categories. This performance substantiates the effectiveness of our approach, validating the hypothesis that using an MLP-based FAI model to identify false positives within an NF model’s positive predictions through semantic features is a viable strategy. A noteworthy observation is that the FAI model exhibits more pronounced improvements in the Fastflow model, which aligns with the findings presented in Section 4.3. This enhanced performance in Fastflow is attributed to the model generating a greater number of positive predictions in most scenarios and the false-positive patches produced by Fastflow possessing more distinct size-related differentiating characteristics, thus making them more amenable to the FAI model’s utilization of semantic features.

Table 4 presents image-level performance metrics, which do not exhibit as pronounced variations as the pixel-level metrics. The introduction of the FAI module leads to minor improvements in the image-level AUROC for the Cflow model in the Cable, Pill, Screw, and Zipper categories, while the Capsule and Wood categories experience slight reductions. The Fastflow model shows mixed results, with decreases in six categories and improvements in five categories. In terms of the image-level F1-score, the FAI module either maintains or marginally decreases performance for both models.

A notable observation is that in some cases in which experiments are conducted on individual categories, despite an increase in pixel-level metrics, there is a decrease in image-level performance. This phenomenon is particularly evident in the Fastflow model’s experiments on the Screw category. This behavior can be attributed to the logic of our method, which individually assesses positive patches at the object level to adjust image-level prediction scores. It represents a process in which quantitative changes result in qualitative shifts. According to the logical framework of our approach, when a significant area of false-positive patches is detected in an image, it leads to a notable reduction in the image-level prediction score. The baseline models inherently exhibit differing score distributions for image-level prediction, significantly influenced by the choice of the image-level anomaly score factor $r_{i}$. Therefore, the selection of this parameter becomes a dilemma when positive samples are scarce. A value that is too high may adversely affect small-area positive samples during the test phase, as seen in Fastflow’s Screw category, while an excessively low value tends to be conservative, reducing the FAI model’s impact on image-level performance.

In summary, the FAI model effectively reduces false positives at the pixel level and enhances classification predictions across more categories.

#### 4.4.2. Visualization of Segmentation Results

**Figure 7:**
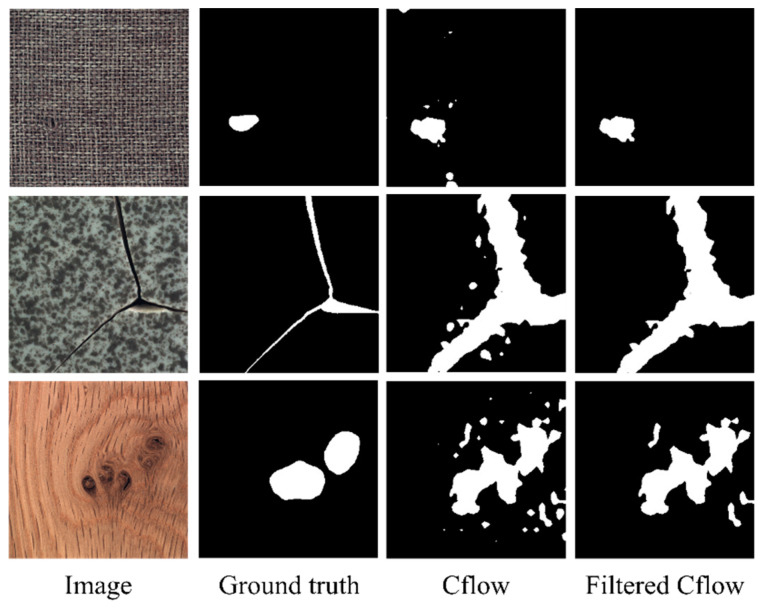
Visualization of the detection results of Cflow on MVTec AD dataset categories: carpet, tile, and wood.

**Figure 8:**
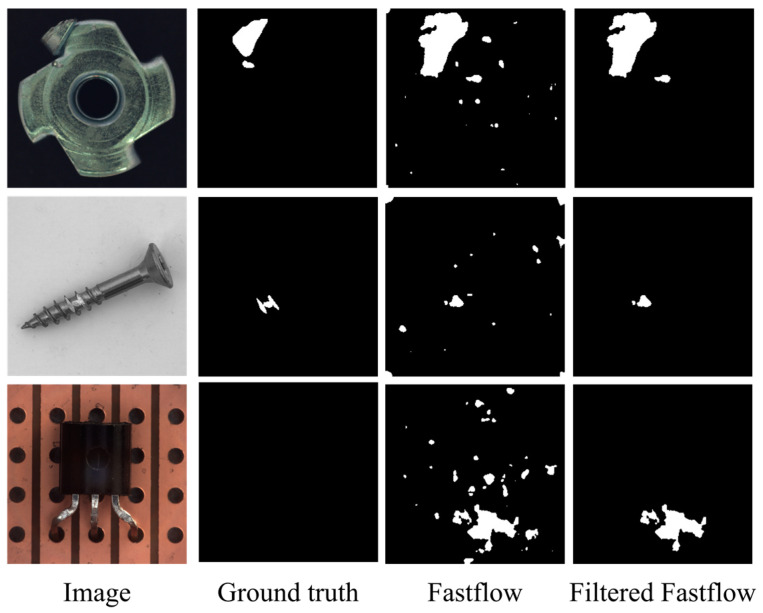
Visualization of the detection results of Fastflow on MVTec AD dataset categories: metal nut, screw, and transistor.

Furthermore, we incorporated illustrative images from our experiments to visually demonstrate the substantial reduction in false positive predictions by our FAI model when applied to baseline models. Importantly, this reduction is achieved without actual anomaly samples, all while preserving true positive predictions. Specifically, Figure 7 and Figure 8 present segmentation results for the Cflow and Fastflow models on three categories from the MVTec AD dataset, offering a more intuitive depiction of the efficacy of our proposed approach.

In Figure 7, we observe that the Cflow model exhibits relative robustness, albeit with some false-positive predictions, albeit in smaller quantities compared to Fastflow, as evident in Figure 8. Notably, the FAI model effectively eliminates a majority of false-positive patches while preserving true positive pixels. This observation not only explains the improvement in pixel-level performance, as highlighted in Table 3 but also affirms the effectiveness of our approach, which leverages object-level semantic features to eliminate false-positive patches.

Figure 8 illustrates the performance of Fastflow on several representative images. It is evident that the FAI model successfully identifies a substantial number of false-positive patches. We intentionally selected the “screw” category, which has been a subject of debate in terms of image-level performance, to elucidate the reason for the observed degradation in image performance. In the screw category experiments, the size of the objects in the images is limited, which inherently restricts the area available for true positive predictions. However, the false alarms generated by Fastflow extend beyond screw region and are spread throughout the background. In such cases, employing the same image-level anomaly score factor as used in experiments on other categories results in overly reduced scores for images containing anomalies, leading to the problem of true positive predictions being modified into negative predictions at the image level. Therefore, in scenarios in which the potential defect area is relatively small, it may be advisable to use a more conservative parameter setting.

### 4.5. Experimental Results on Freight Train Side Frame Fault Detection

#### 4.5.1. Anomaly Maps on the Training Datasets

**Figure 9:**
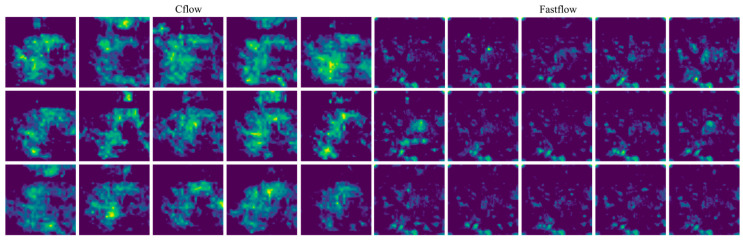
Anomaly maps of Cflow (**left**) and Fastflow (**right**) on the anomaly-free TFDS-SF training dataset.

Figure 9 displays anomaly maps for TFDS inspection, showcasing different characteristics from the previous scenario. In this application, Fastflow exhibits superior pixel-level performance as its anomaly map scores are closer to 0 (depicted in dark blue), representing the ideal situation. It is worth noting that Cflow appears sensitive to light reflections from certain material components within the image, indicating potential errors in its predictions.

Given the complex environmental factors and varying component lifetimes, both baseline detectors underperform on the TFDS-SF dataset. They fail to identify all defects and generate a few false alarms at the image level. These results align with the analysis provided in Section 3.1, suggesting that false alarms stem from adverse factors. In this context, false positives are related to background disturbances, component reflection variations due to different outdoor operating times, distinct imaging effects from light source differences, and dirt on the side frame surface. Cflow performs better at the image level, while Fastflow excels in pixel-level performance metrics.

#### 4.5.2. Performance Comparisons

As indicated in Table 5, our proposed filtering method significantly improves the performance of the built-in Cflow, enhancing pixel metrics by 9.69% and 212%. While the image AUROC of Cflow improves, the image F1-score remains consistent. This suggests that the FAI model’s reductions at the image level are accurate, but their magnitudes are not substantial enough to correct classification outcomes. Fastflow’s image-level vulnerability primarily arises from its image-level prediction score normalization strategy. Furthermore, Fastflow continues to generate minor false alarm regions at the pixel level. The FAI algorithm effectively eliminates these regions, resulting in substantial improvements in four performance metrics, with rates of 17.59%, 30.68%, 0.76%, and 55.82%.

#### 4.5.3. Visualization of Segmentation Results

**Figure 10:**
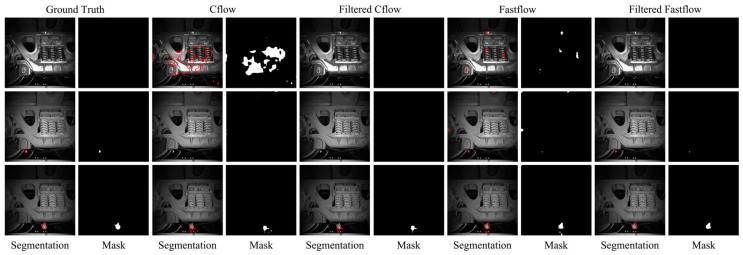
Pixel-level fault detection results of Cflow (**medium**) and Fastflow (**right**) on freight train side frame fault detection: segmentation boundaries and masks.

As shown in Figure 10, the false positive predictions generated by Cflow and Fastflow exhibit semantic characteristics that resemble the anomaly maps of the training images in Figure 9. Cflow is significantly affected by metal reflections, leading to extensive false-positive predictions, whereas this interference is less pronounced in Fastflow. However, Fastflow tends to produce some small false alarm regions near the image edges. These observations align with the assumption presented in our FAI method.

Visualizations of the detection results in the TFDS images reveal the FAI model’s effective filtration of false alarm regions while preserving defect regions, particularly in the case of Fastflow. An intriguing scenario emerges in the second row of the visualizations. The original image features an undetected defect by Cflow, while Cflow erroneously identifies small upper regions as defects, resulting in false alarms. The FAI model successfully eliminates these false alarms, yet the filtered image-level prediction score remains high. Consequently, the defect mask becomes null, even though the prediction for this image is a defect. This observation underscores the efficacy of our image-level operation following the filtration process.

By adjusting in proportion to the eliminated area ratio, the FAI model effectively retains its image-level discriminative capability.

### 4.6. Parameter Analysis on Semantic Feature Selection and Binarization Thresholds

Previous experiments involved the comparison of baseline detectors’ performance with and without the FAI model in a fixed configuration. Table 6 presents the results of experimental parameter studies conducted on two aspects of the FAI algorithm: the discriminative physical constraints $x_{m}$ of the filter (Section 3.3) and the quantity of binarization thresholds Q in sample synthesis (Section 3.4).

As displayed in Table 6, the inherent performance of both trackers improves across all four parameter settings. These findings underscore the positive impact of the FAI model. In particular, changes in the quantity of binarization thresholds yield greater improvements compared to the inclusion of additional physical attributes. Notably, substantial enhancements across multiple binarization thresholds suggest that the proposed sample synthesis method is influenced by the specific characteristics of the detector.

We enrich the $x_{m}$ setting with additional physical attributes derived from the histogram of pixel intensity ratios (a grayscale image with a four-bin histogram), expanding the input vector dimension to m=8. It is evident that the inclusion of the four-bin histogram of intensities, representing image texture information, further enhances the performance of the FAI model. In this context, Fastflow benefits more from this influence in terms of image-level detection metrics. The marginal improvements observed between m=4 and m=8 illustrate the discriminative capacity of different attribute combinations for distinguishing between false positives and true positives.

Regarding the binarization threshold settings, the two NF models exhibit more pronounced performance differences. The Q = 9 setting utilizes binarization thresholds spanning from 0.4 to 0.52, while to assess the impact of binarization threshold quantities, we consider the scenario with Q = 1, where the threshold τ=0.5. The substantial improvements observed between the built-in, Q = 1, and Q = 9 settings underscore the effectiveness of the sample synthesis strategy within the FAI model.

## 5. Conclusions

This study underscores the potential for normalizing flow defect detectors to achieve substantial performance enhancements by acquiring insights from the anomaly-free training dataset.

We propose a highly efficient two-stage approach aimed at elevating the defect performance of unsupervised anomaly detectors through semantic features. Specifically, our methodology introduces a multilayer-perceptron-based FAI algorithm which is adept at incorporating semantic features associated with discriminative physical constraints at the object level. Furthermore, to extend the applicability of our approach to a broader range of scenarios, we devise a training sample synthesis strategy that allows the FAI model to generate training samples tailored to the characteristics of the baseline detector.

To empirically evaluate the effectiveness of our optimization approach, we conduct extensive experiments on two state-of-the-art normalizing flow algorithms across a diverse set of industrial applications. The results from these experiments provide compelling evidence of significant improvements across all evaluated metrics.

In summary, our research not only contributes to the advancement of normalizing-flow-based defect detection but also offers practical insights and solutions for enhancing anomaly detection performance in real-world computer vision applications.

## Figures and Tables

**Table 1:** Experimental settings of the baseline normalizing flow models.

Model	Backbone	Pretrained	Image Size	Learning Rate	Stop Patience	Decoder	Flow Steps
Cflow	Wide_resnet50_2	True	256	0.0001	2	freia-cflow	-
Fastflow	Resnet18	True	256	0.001	3	-	8

**Table 2:** Performance comparisons on the bottle quality inspection scene.

Performance Metric	Cflow (%)	Cflow + FAI (%)	Fastflow (%)	Fastflow + FAI (%)
Image AUROC	100.00	100.00	100.00	100.00
Image F1-score	100.00	100.00	97.67	**100.00**
Pixel AUROC	98.34	**98.37**	97.94	**97.99**
Pixel F1-score	71.96	**72.08**	62.32	**63.40**

**Table 3:** Pixel-level performance comparisons on the MVTec AD dataset with the format (pixel-level AUROC; pixel-level F1-score).

Categories	Cflow (%)	Cflow + FAI (%)	Fastflow (%)	Fastflow + FAI (%)
Cable	(96.09, 53.45)	(**96.11**, **53.79**)	(96.22, 54.45)	(**96.36**, **57.13**)
Capsule	(98.81, 49.37)	(**98.82**, **49.66**)	(98.51, 44.80)	(**98.53**, **45.95**)
Carpet	(98.64, 62.85)	(**98.65**, **63.33**)	(98.14, 58.26)	(**98.16**, **59.25**)
Grid	(96.52, 32.91)	(**96.53**, **33.15**)	(98.24, 43.65)	(**98.25**, **44.44**)
Hazelnut	(98.57, 58.41)	(**98.58**, **58.49**)	(95.72, 50.94)	(**95.75**, **51.60**)
Leather	(99.67, 61.23)	(99.67, **61.40**)	(99.62, 56.99)	(99.62, **57.16**)
Metal nut	(98.05, 80.81)	(**98.06**, **81.00**)	(96.64, 72.32)	(**96.74**, **73.57**)
Pill	(98.62, 73.00)	(**98.63**, **73.34**)	(97.53, 63.13)	(**97.60**, **64.77**)
Screw	(97.84, 32.57)	(**97.85**, **33.69**)	(92.48, 7.17)	(**93.10**, **14.12**)
Tile	(96.39, 65.75)	(**96.44**, **66.36**)	(93.33, 55.80)	(**93.45**, **56.70**)
Toothbrush	(98.29, 47.73)	(98.29, **95.76**)	(97.43, 47.22)	(**97.50**, **49.02**)
Transistor	(95.76, 57.35)	(**95.79**, **57.69**)	(95.93, 52.14)	(**96.10**, **54.60**)
Wood	(94.85, 54.45)	(**94.87**, **54.68**)	(95.85, 57.29)	(**95.88**, **57.68**)
Zipper	(98.06, 57.03)	(**98.07**, **57.47**)	(97.47, 50.47)	(**97.60**, **53.45**)

**Table 4:** Image-level performance comparisons on the MVTec AD dataset with the format (image-level AUROC; image-level F1-score).

Categories	Cflow (%)	Cflow + FAI (%)	Fastflow (%)	Fastflow + FAI (%)
Cable	(95.11, 91.19)	(**95.16**, 91.19)	(91.59, 84.36)	(**92.19**, **85.17**)
Capsule	(**95.85**, 95.41)	(95.81, 95.41)	(**88.19**, 92.86)	(87.99, 92.86)
Carpet	(97.99, 96.05)	(97.99, 96.05)	(98.56, 95.51)	(98.56, **96.05**)
Grid	(91.98, 90.00)	(91.98, 90.00)	(98.16, 97.39)	(98.16, 97.39)
Hazelnut	(99.96, 98.57)	(99.96, 98.57)	(79.25, 86.62)	(**79.36**, 86.62)
Leather	(100.00, 99.45)	(100.00, 99.45)	(99.97, 99.46)	(**100.00**, 99.46)
Metal nut	(99.66, 99.46)	(99.66, 99.46)	(**95.36**, 92.31)	(95.06, **92.78**)
Pill	(95.36, 96.43)	(**95.53**, 96.43)	(91.43, 91.21)	(91.63, 91.21)
Screw	(80.65, 84.98)	(**80.84**,84.98)	(**72.66**, **86.89**)	(52.57, 46.34)
Tile	(100.00, 98.82)	(100.00, 98.82)	(**93.76**, 90.36)	(93.65, 90.36)
Toothbrush	(85.28, 86.96)	(85.28, 86.96)	(80.83, 82.54)	(**81.11**, **83.87**)
Transistor	(99.33, 91.89)	(99.33, 91.89)	(**90.00**, 70.59)	(89.38, **75.95**)
Wood	(**98.68**, 97.52)	(98.42, 97.52)	(97.46, 98.33)	(**97.63**, 98.33)
Zipper	(93.67, 96.36)	(**94.01**, 96.36)	(**92.07**, **95.55**)	(91.81,94.69)

**Table 5:** Performance comparisons on freight train side frame fault detection.

Performance Metric	Cflow (%)	Cflow + FAI (%)	Fastflow (%)	Fastflow + FAI (%)
Image AUROC	62.82	**68.91**	58.33	**68.59**
Image F1-score	37.21	37.21	34.78	**45.45**
Pixel AUROC	93.14	**96.90**	98.77	**99.52**
Pixel F1-score	10.39	**32.44**	37.91	**59.07**

**Table 6:** Performance comparisons of parameter studies on freight train side frame fault detection.

Detector	Metric	Built-In	With FAI			
			Q1m4	Q1m8	Q9m4	Q9m8
Cflow	Image AUROC (%)	62.82	63.14	64.10	68.91	69.55
	Image F1-score (%)	37.21	37.21	38.10	37.21	38.10
	Pixel AUROC (%)	93.14	94.07	95.01	96.90	97.10
	Pixel F1-score (%)	10.39	16.40	17.88	32.44	33.18
Fastflow	Image AUROC (%)	58.33	58.97	60.58	68.59	69.87
	Image F1-score (%)	34.78	38.10	40.01	45.45	47.62
	Pixel AUROC (%)	98.77	99.15	99.16	99.52	99.53
	Pixel F1-score (%)	37.91	54.40	55.71	59.07	60.01

## Data Availability

Data are contained within the article.
